# Scalp eschar and neck lymph adenopathy after a tick bite (SENLAT) in Tuscany, Italy (2015–2022)

**DOI:** 10.1007/s15010-023-02079-8

**Published:** 2023-08-11

**Authors:** Anna Barbiero, Tommaso Manciulli, Michele Spinicci, Iacopo Vellere, Maria Grazia Colao, Gian Maria Rossolini, Alessandro Bartoloni, Didier Raoult, Lorenzo Zammarchi

**Affiliations:** 1 Department of Experimental and Clinical Medicine, University of Florence, 50134 Florence, Italy; 2 Department of Infectious and Tropical Diseases, Careggi University Hospital, 50134 Florence, Italy; 3 Tuscany Regional Referral Center for Tropical Diseases, Careggi University Hospital, 50134 Florence, Italy; 4 Clinical Microbiology and Virology Unit, Careggi University Hospital, 50134 Florence, Italy; 5 Consulting Infection Marseille SAS, 16 rue de Lorraine, 13008 Marseille, France

**Keywords:** Rickettsiaceae infections, Tick-borne diseases, Dermacentor, Zoonosis, SENLAT

## Abstract

**Introduction:**

The Scalp Eschar and Neck Lymph Adenopathy After a Tick Bite (SENLAT) syndrome is frequently caused by *Rickettsia slovaca* and *Rickettsia raoultii*. Only six microbiologically confirmed SENLAT cases have been reported in Italy between 1996 and 2021. We report ten cases of SENLAT seen between 2015 and 2022 in a tertiary care center in Tuscany, Italy.

**Cases presentation:**

All patients were women; most common symptoms were scalp eschar on the site of tick bite (100%) and cervical lymphadenopathy (90%). No microbiological identification was obtained. Persistent alopecia, for several months to years, was observed in four patients. The known difficulty of microbiological diagnosis in SENLAT was worsened, in our cases, by factors as the absence of ticks available for identification and microbiological study, and antibiotic treatment administration previous to microbiological tests.

**Conclusion:**

The report highlights the presence of SENLAT in Italy, aiming to raise the awareness toward the emergence of this clinical entity.

**Supplementary Information:**

The online version contains supplementary material available at 10.1007/s15010-023-02079-8.

## Background

The syndrome currently known as Scalp Eschar and Neck Lymph Adenopathy After a Tick Bite (SENLAT) has a history made of several changes in denomination.

*Rickettsia slovaca* was firstly isolated in *Dermacentor* spp. tick in 1968 in Slovakia and for several years it was believed not to have any pathogenic role for humans [1]. In 1987, in the Hungarian center for tick-borne diseases, a first case of tick bite on the scalp characterized by appearance of eschar and neck lymphadenopathy was described and many other cases followed. Between 1996 and 1997 this syndrome was termed Tick-borne lymphadenopathy (TIBOLA) by the research group of the Centre National de Référence des Rickettsies, Coxiella et Bartonella, Marseille, France, and it was associated to the isolation of *R. slovaca* and identification of *Dermacentor* ticks as vectors [2, 3]. In 2003 *R. slovaca* was finally isolated in a patient [4]. After being named DEBONEL (*Dermacentor*-born necrosis, erythema and lymphadenopathy) [5], the last change in terminology was once more introduced by the Marseille group, where the disease was defined as SENLAT, in order to define a clinical entity that is not associated to a precise microbiological diagnosis [6], characterized by a tick bite on the scalp followed by appearance of an eschar on the bite site and neck lymphadenopathy. Other common symptoms are fever, myalgia, neck pain, headache. Appearance of facial edema has been reported, while macular lesions of the extremities or multiple eschars are rare findings [7]. It usually presents as a mild rickettsiosis, but severe forms have been described and prolonged symptoms are reported, mostly if the illness is not appropriately treated [8, 9]. Most common sequelae include alopecia on the eschar site, and chronic asthenia, lasting up to several months [10]. The most common causative agents are *R. slovaca* and *Rickettsia raoultii*, but other pathogens have been identified in SENLAT patients, such as *Rickettsia massiliae* [11, 12], *Rickettsia rioja*, *Rickettsia sibirica mongolitimonae*, *Bartonella henselae*, *Coxiella burnetii*, *Borrelia burgdorferi* and *Francisella tularensis* [13]. No specific clinical pattern has emerged in relation to particular pathogens. Diagnosis of SENLAT is based on clinical-epidemiological features given that no microbiological test can reliably identify all potential pathogens involved in its manifestations. As a consequence, it may be hard to recognize and diagnose this disease. *Dermacentor spp*. ticks appear to be the most frequently involved vector: *Dermacentor marginatus* is spread all over Mediterranean areas of Europe and North Africa, while *Dermacentor reticulatus* is more commonly found in Central and Western Europe and in some areas of Russia [14]. *Dermacentor* adult ticks are usually active from March to April and from September to October. They climb onto grasses or bushes and wait for their hosts at an height of 0.5–1.5 m [15]. Their biting habits include wild hairy mammals and, among humans, they prefer biting women and children on hairy areas of the body, frequently in the scalp [16]. SENLAT is one of the most common rickettsiosis in Europe, second only to Mediterranean Spotted Fever, and it has been reported in Italy, Spain, France, Hungary, Bulgaria, Portugal [7]. Raoult et al. reported that 19% of tick-borne diseases in Europe are associated with *R. slovaca* and between 1 and 17% of *Dermacentor* ticks are reported to be positive for *R. slovaca* in Europe [2]. According to a systematic literature review, only six microbiologically confirmed cases of SENLAT have been reported in Italy from 1996 to 2021 [16]; in these cases, *R. slovaca* and *R. massiliae* were identified with molecular methods as the causative agents [10–12, 17]. Moreover, *R. slovaca*, and sporadically *R. raoultii*, positive *Dermacentor* ticks were found in Piedmont, Liguria, Sardinia, Tuscany and Abruzzo in wild boars, possibly representing a relevant wild reservoir with a potential role in the eco-epidemiology of rickettsiosis in these regions [18, 19]. We present ten cases of SENLAT seen at a referral center in Central Italy, discussing the clinical characteristics and diagnostic features of our cases.

## Materials and methods

The Infectious and Tropical diseases department of Careggi University Hospital is located in Florence, Tuscany, central Italy, and includes a regional referral center for tropical and vector-borne diseases. We searched the records of our center for patients seen between January 1st 2015 and May 31st 2022, who presented with symptoms compatible with SENLAT. Data on demographic variables, epidemiological information, clinical data, comorbidities or special circumstances (e.g., pregnancy), laboratory findings, microbiological tests, treatment and outcomes were analyzed.

### Microbiological investigations

Serological tests were performed at our laboratory with a commercial kit for *Rickettsia conorii* IgG (Indirect Immunofluorescence, *R. conorii* IgG IFA^®^, Fuller Laboratories, California, U.S.A., available until April 2021; Chemiluminescent immunoassay, Virclia *R. conorii* IgG CLIA^®^, ALIFAX, Padua, Italy, available from May 2021 and still being used), *Rickettsia typhi* IgG (*R. typhi* IgG IFA^®^, Fuller Laboratories, California, U.S.A.), *B. henselae* IgG-IgM (IFA, *B. henselae* IgG and *B. henselae* IgM^®^, EUROIMMUN, Padua, Italy). Enzyme-linked immuno-assay (ELISA, Enzygnost *Borrelia* IgG^®^, Enzygnost Borrelia IgM^®^, Siemens Healthcare, Milan, Italy) was performed for *Borrelia* IgG-IgM until 2021, CLIA (LIAISON *Borrelia* IgG^®^, LIAISON *Borrelia* IgM^®^, DiaSorin, Vercelli, Italy) was available for Borrelia IgG-IgM tests afterwards. When possible, additional samples as scalp eschars, lesion swabs, whole blood and serum samples, were preserved at − 80 °C after collection and shipped to the Centre National de Référence des Rickettsies, Coxiella et Bartonella, Marseille, France, for further microbiological investigation. In the Marseille specialized center the following investigations were performed: real-time PCR addressing Spotted Fever Group (SFG) *Rickettsiae*, *Leishmania* spp, 16S RNA amplicon sequencing, Marseille virus, *Anaplasma* spp, *Bartonella* spp, *B. burgdorferi* sensu lato, *C. brunetii*, *Coxiella* spp, *Francisella* spp, *S. aureus*, *S. pyogenes*, *T. pallidum*, Orthopoxvirus, Parapoxvirus on scalp eschars, lesion swabs and blood samples; IFA serological assays for *R. slovaca* IgG-IgM, *R. conorii* IgG-IgM, *R. typhi* IgG IgM, *C. burnetii* IgG-IgM, *Rickettsia felis* IgG-IgM, *B. henselae* IgG, *Bartonella quintana* IgG. For two patients, a swab culture for common fungal and aerobic and anaerobic pathogens was performed. Further details about the tests performed in the Centre National de Référence des Rickettsies, Coxiella et Bartonella have previously been described [20, 21]. When possible, seriated serum samples were obtained over time in order to check for seroconversion.

## Cases presentation

Our search found ten patients with SENLAT-compatible manifestations. Information about demographic, clinical, microbiological, treatment and outcome features are reported in Table 1.

**Table 1:** The table resumes main epidemiological and clinical features, microbiological tests, treatments and outcomes of the described cases

	Patient 1	Patient 2	Patient 3*	Patient 4	Patient 5	Patient 6	Patient 7	Patient 8	Patient 9*	Patient 10
Sex	Female	Female	Female	Female	Female	Female	Female	Female	Female	Female
Age	55	40	31	73	50	30	59	56	41	31
Municipality of tick bite (and province)	Fiesole (Florence)	Pistoia (Pistoia)	Palaia (Pisa)	Casentino (Arezzo)	Palazzuolo sul Senio (Florence)	Vinci (Florence)	Compiobbi (Florence)	Valdibure (Pistoia)	Serpiolle (Florence)	Mugello (Florence)
Presumed timing of tick bite (symptoms onset = day 0)	April 2015 (day -10)	May 2017 (day -7)	April 2019 (day -14)	April 2019 (day -1)	April 2019 (day -12)	May 2021 (day -6)	April 2021 (day -7)	March 2022 (day -7)	April 2022 (day -3)	May 2022 (day -10)
Symptoms (symptoms onset = day 0)	Fever (39 °C), LA, vertigo, nausea, facial edema (day 0). Scalp eschar (day 2).	Fever (38.5 °C), lymphangitis (day 0). Multiple scalp eschars (day 3).	LA (day 0). Fever (37.6 °C), scalp eschar (day 2).	Fever (37.3 °C), LA (day 0). Scalp eschar (day 7).	Fever (37.5 °C), LA (day 0). Scalp eschar (day 2).	LA (day 0). Fever (38.7 °C), multiple scalp eschars (day 3).	LA (day 0). Fever (37.5 °C), scalp eschar (day 5). Facial edema (day 7).	LA (day 0). Scalp eschar (day 3).	LA (day 0). Scalp eschar (day 1).	LA (day 0). Fever (37.7 °C), scalp eschar (day 2).
Direct microbiological tests	–	– Lesion swab culture – Lesion swab PCR^§^ – Blood PCR^§^	– Blood PCR for SFG *Rickettsiae*	– Lesion swab PCR^§^ – Blood PCR^§^	– Eschar PCR^§^ – Lesion swab PCR^§^ – Blood PCR^§^	– Eschar PCR for SFG *Rickettsiae* – Lesion swab culture – Lesion swab PCR for SFG *Rickettsiae* – Blood PCR for SFG *Rickettsiae*	–	–	–	–
Serological tests	*R. conorii* IgG IgM *R. typhi* IgG IgM *C. brunetii* IgG IgM *R. felis* IgG IgM *B. henselae* IgG	*R. slovaca* IgG IgM	*B. henselae* IgG *B. burgdorferi *sensu lato IgG *Francisella* spp. total Ig *R. conorii* IgG *R. typhi* IgG	*B. burgdorferi* sensu lato IgG IgM *R. conorii* IgG *R. typhi* IgG *R. slovaca* IgG IgM	*R. slovaca* IgG IgM	*R. conorii* IgG	*R. conorii* IgG *B. burgorferi* sensu lato IgG IgM	*B. burgorferi* sensu lato IgG IgM	*B. burgorferi* sensu lato IgG IgM	*R. conorii* IgG
Antibiotic treatments (starting day, duration)	-Doxy (day0, 21 days) -A/C (day2, 5 days)	-A/C (day0, 5 days) -Doxy (day3, 14 days)	–Azithro (day 2, 5 days)** -A/C (day9, 10 days)	-A/C (day0, 6 days)	–Azithro (day-5, 3 days)** -Doxy (day5, 14 days)	-Doxy (day 3, 14 days)	-A/C (day1, 21 days) -Doxy (day6, 2 days)***	-Doxy (day3, 14 days)	-A/C (day3, 7 days)	-Doxy (day10, 7 days)** -Azithro (day17, 3 days)
Persistent symptoms	Eschar (until day 21) Alopecia (still present)	Eschar (until day 21) Alopecia (until day 120)	Eschar (until day 60) Alopecia (until day 365)	Lost at follow-up	Eschar (until day 45)	Eschar (until day 30)	Eschar (until day 21)	Not available	Eschar (until day 28) Alopecia (still present)	Eschar (until day 15)

Onset of symptoms is considered as “day 0”, and timing of events is in relation to “day 0”

*LA* lymphadenopathy; *A/C *amoxicillin/clavulanate; *Doxy *doxycycline; *Azithro* azithromycin

*Specific condition: pregnant

**Unsuccessful/intolerable treatment

***Treatment interrupted for suspected allergic reaction

^§^PCR tests were addressed to: SFG *Rickettsiae*, *Leishmania* spp, 16S RNA, Marseille virus, *Anaplasma* spp, *Bartonella* spp, *B. burgdorferi *sensu lato, *C. brunetii, Coxiella* spp, *Francisella* spp, *S. aureus*, *S. pyogenes*, *T. pallidum*, Orthopoxvirus, Parapoxvirus

### Demographic and exposure locations

All patients were women between 30 and 73 years (median age was 45.5 years); two of them were pregnant. All patients were bitten between March and May during outdoor activities in rural areas located in Tuscany, with the geographical distribution represented on supplementary materials, Fig. 1. All patients denied recent travelling abroad and they all reported to have found a single tick attached in the scalp.

**Fig. 1:**
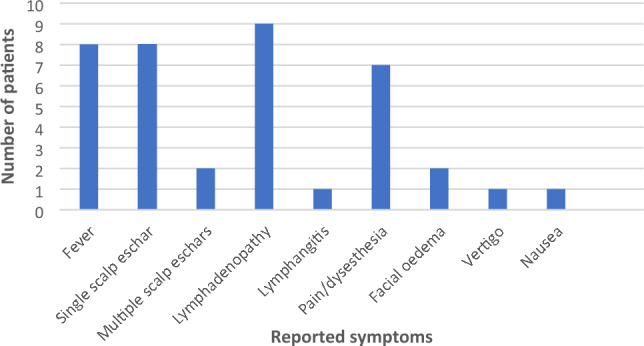
Frequency of main symptoms reported by patients

### Clinical presentations and evolution

**Fig. 2:**
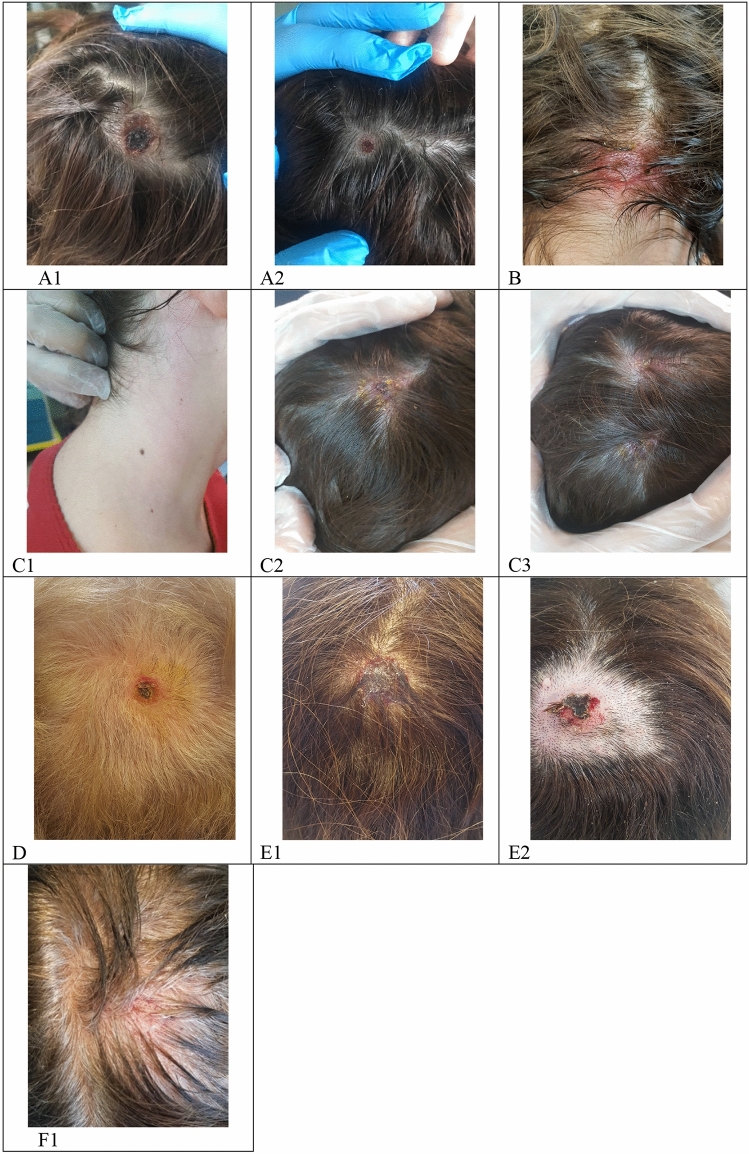
**A1, A2.** Patient 1, eschars at day 9; **B.** Patient 8, eschar at day 6; **C1.** Patient 5, lymphangitic stria at day 4; **C2, C3.** Patient 5, scalp eschars at day 4; **D.** Patient 4, eschar at day 31; **E1, E2.** Patient 6, eschar at day 23 before (E1) and after (E2) debridement and medication; **F1.** Patient 7, eschar after 18 days

Time between presumptive date of bite and beginning of symptoms went from 1 to 14 days (median time 7 days). According to reported presumptive dates of tick bites, tick attachment duration ranged between 1 and 10 days (median time 3 days), as showed on supplementary materials, Fig. 2. As shown in Fig. 1, eight patients presented with fever (median maximum temperature was 37.7 °C), which lasted for a median time of 2.5 days. Cervical lymphadenopathy was reported by nine patients, while one patient presented with a lymphangitic stria. Eight patients presented with scalp eschar on the site of tick bite and two patients presented with multiple scalp eschars. Scalp eschars were reported to appear from 1 to 7 days after onset of lymphadenopathy. Eight patients reported severe pain and dysesthesia either on the site of tick bite or on the lymph nodes area, in some cases irradiating to the whole scalp. Two patients presented with facial edema, which regressed after administration of steroids and/or non-steroid anti-inflammatory agents; only one patient reported nausea and vertigo. Disseminated rash was not observed. Photographic documentation of the visible lesions is illustrated in Fig. 2.

### Microbiological and laboratory investigations

Laboratory findings were normal in most cases: one patient presented with mild leukocytosis (up to 12.9 × 10^9/L, patient 6); three patients had a mild increase in C reactive protein (up to 37 mg/L, 32 mg/L and 32 mg/L in the case of patient 6, patient 1 and patient 7, respectively), recorded between 1 and 6 days after symptoms onset and followed by normalization during the second week after symptoms onset. No cases of renal or hepatic function impairment were recorded. At our laboratory, serological tests for *R. conorii* IgG, *R. typhi* IgG, *B. henselae* IgG-IgM, *B. burgdorferi* sensu lato IgG-IgM were performed.

Additional microbiological investigations were conducted at the Centre National de Référence des Rickettsies, Coxiella et Bartonella, Marseille, France. Microbiological tests performed for each patient are reported in Table 1. It was possible to perform PCR on scalp eschar biopsies (2/10 patients), blood (5/10), lesion swabs (4/10). In all cases, all performed real time PCR on blood and lesions samples and cultures resulted negative. For what concerns serologies, performed either in our laboratory, in Marseille laboratory, or both: 3 patients were tested at presentation only, 4 were tested in convalescent phase (median latency 35 days) and 3 patients were tested both at presentation and in the convalescent phase. All serological tests were negative.

Of note, all patients came to our center only after visiting another doctor (general practitioners or emergency department specialists) and had removed the tick before the visit to our center without preserving it, therefore it was not possible to perform taxonomic identification or microbiological investigation of the tick. Moreover, they all started antibiotic treatment before any microbiological investigation was performed. Timelines representing main events in the clinical history of all patients are represented in supplementary materials, Fig. 2.

### Treatment

Five patients recovered after treatment with doxycycline and one was successfully treated with azithromycin. Three patients were treated with amoxicillin/clavulanate before our evaluation and came to our center after symptoms regression in two cases, therefore no further treatment was given; in the third case, who presented with persistent symptoms, doxycycline was administered but it was then interrupted due to suspected allergic reaction. Two patients, initially treated with azithromycin in one case and doxycycline in the other, required additional treatment with amoxicillin/clavulanate in the suspicion of local superinfection (Table 1). Three patients, presenting with large scalp eschars (> 3 cm in diameter), that is patient 4, patient 5 and patient 6, required repeated wound dressing in addition to systemic antibiotic treatment.

### Outcomes

Scalp eschar persisted after treatment in all patients for a median time of 24.5 days, excluding patient n. 4 which was lost at follow-up and patient n. 8 who has not come back to visit by the time the manuscript was written. Duration of persistent alopecia in the eschar site for each patient is reported in Table 1.

## Discussion

Clinical and epidemiological features of these cases are coherent with SENLAT. Tick attachment duration ranged between 1 and 10 days, while symptoms appeared between 1 and 14 days after the presumptive day of tick bite; however, reported days of tick bites were only presumptive and a high grade of imprecision should be considered for these results. Indeed, most patients did not note the happening of the tick bite and could only correlate the presumptive timing of the tick bite with the recent outdoor activities performed in rural areas. Presumptive duration of tick attachment did not seem to correlate with symptoms severity in our cases, however the small number of reported cases does not allow to reach any statistical significance.

Scalp eschar (or multiple scalp eschars) and neck lymphadenopathy were observed in the great majority of our patients, coherently with other findings reported in literature [9, 17]. Interestingly, in all cases, scalp eschar appearance was observed between 1 and 7 days after the onset of the first symptoms (that were usually lymphadenopathy and/or fever); further research is needed to define most typical clinical evolution in SENLAT patients and typical timing of appearance of more frequent symptoms. Moreover, scalp eschars appearance could have passed unnoticed for several days, with consequent high probability of imprecision in describing the timing of their onset.

In our case series, a high percentage of patients reported fever, while this symptom has been reported in a lower proportion of cases by other authors [9, 17]; since the study was conducted in a tertiary care center, a selection bias could partly explain this finding.

Microbiological identifications are missing in our cases. This result could be explained by some factors. First of all, tick examination is very helpful in order to get diagnosis, since the identification of a *Dermacentor* tick, after tick bite on the scalp and associated to typical clinical presentation, is strongly suggestive for SENLAT [2]. Moreover, microbiological study of the tick with molecular tests has an important role in the process of indirect identification of the involved pathogen and has been in some cases the only way to associate SENLAT with a possible pathogen [22]. In our case all patients had removed and thrown the tick before coming to our center, therefore this diagnostic procedure was completely missed.

Molecular diagnosis is the most sensitive way for etiological definition in SENLAT and PCR can be performed either on lesions or lymph nodes biopsies, swabs or blood specimens. However, blood PCR tests do not have high sensitivity due to the localized nature of the disease. It is suggested to perform microbiological investigations as soon as possible after symptoms onset, before any antibiotic treatment and not later than 4 days [22].

In our case, it was impossible to collect early clinical samples. In addition, sensitivity of our microbiological tests was very low due to previously administered antibiotic treatment.

Serologies were negative in all cases; this result is coherent with literature and explained by the fact that serology is not believed to be sensitive in this kind of syndrome, due to its localized nature and evolution [2].

It is worth of mention that 2 out of 10 cases recovered after treatment with amoxicillin/clavulanate alone, which is not active against *Rickettsiae*, suggesting either a natural regression of symptoms independently of antibiotic treatment, or the involvement of a pathogen which is sensible to beta-lactams, possibly as consequence of a localized superinfection.

Absence of pathogen isolation is in line with previously reported cases described in literature: as an example, a review by Pinto et al. reported that only 30% of cases are associated with any microbiological direct identification [14]. This evidence strengthens the hypothesis of presence of still unknown mechanisms and/or pathogens causing the syndrome [2]; in fact, SENLAT has been described as a non-pathogen-specific reaction to *Dermacentor* ticks’ bites or to any microorganism carried by these ticks [9, 21].

Finally, it should be noted that a strong host-parasite association has been demonstrated among wild boars and *D. marginatus* in northern Mediterranean areas, including Tuscany, where 32.1% of tested ticks resulted positive for *R. slovaca* and 1.8% for *R. raoultii*, supporting the possible epidemiological role of these arthropods and relative pathogens in the described cases and, more broadly, in tick borne diseases recorded in this region [18, 23].

## Conclusion

In conclusion, our experience brings attention to presence of SENLAT cases in Italy, which are rarely reported and likely underdiagnosed. Better awareness and microbiological and clinical knowledge of SENLAT could help avoiding misrecognition of this syndrome and improve the knowledge about appropriate treatment and management of this disease. Further studies, ideally conducted on both patients and ticks, are needed both on the clinical aspects of the disease and on its epidemiological features.

### Supplementary Information

Below is the link to the electronic supplementary material.

Supplementary file1 (DOCX 958 KB)

## Data Availability

Data is available from the authors upon reasonable request.
