# Supplementary material for: Scalp eschar and neck lymph adenopathy after a tick bite (SENLAT) in Tuscany, Italy (2015–2022)
Source: Infection. 2023 Aug 11;51(6):1847–54. doi: 10.1007/s15010-023-02079-8 (PMC10665257; doi:10.1007/s15010-023-02079-8)
Supplement: Supplementary file 1 — Supplementary file1 (DOCX 958 KB) [file 15010_2023_2079_MOESM1_ESM.docx]

SUPPLEMENTARY MATERIALS


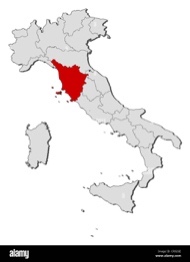


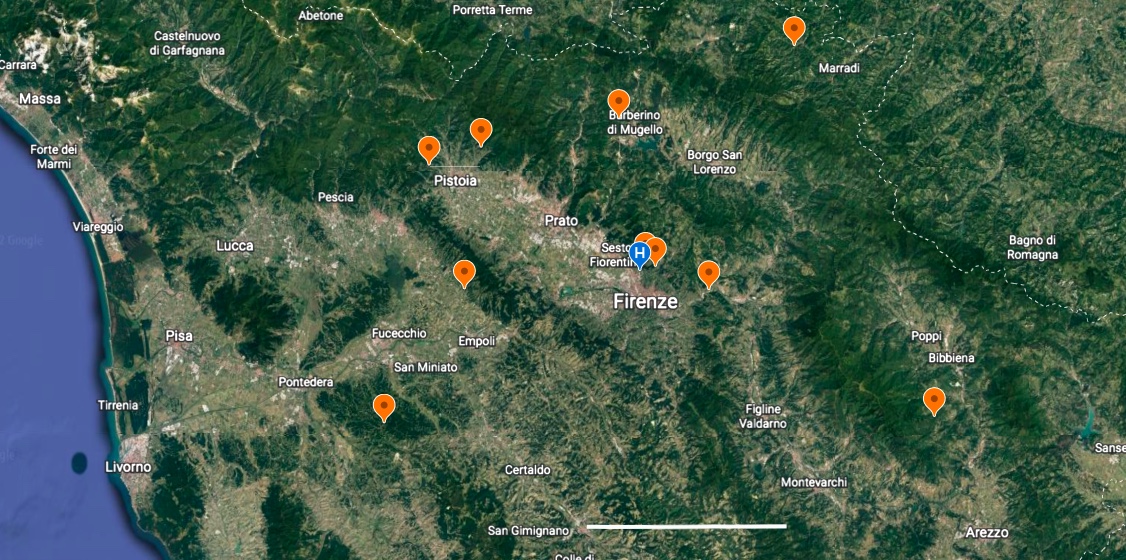


**30km**

30km

**Fig 1**. Tuscany region is represented by the red area in the right-upper image of the Italian peninsula; approximative areas of tick bites are indicated by the red pins. The blue pin, points Careggi University Hospital.


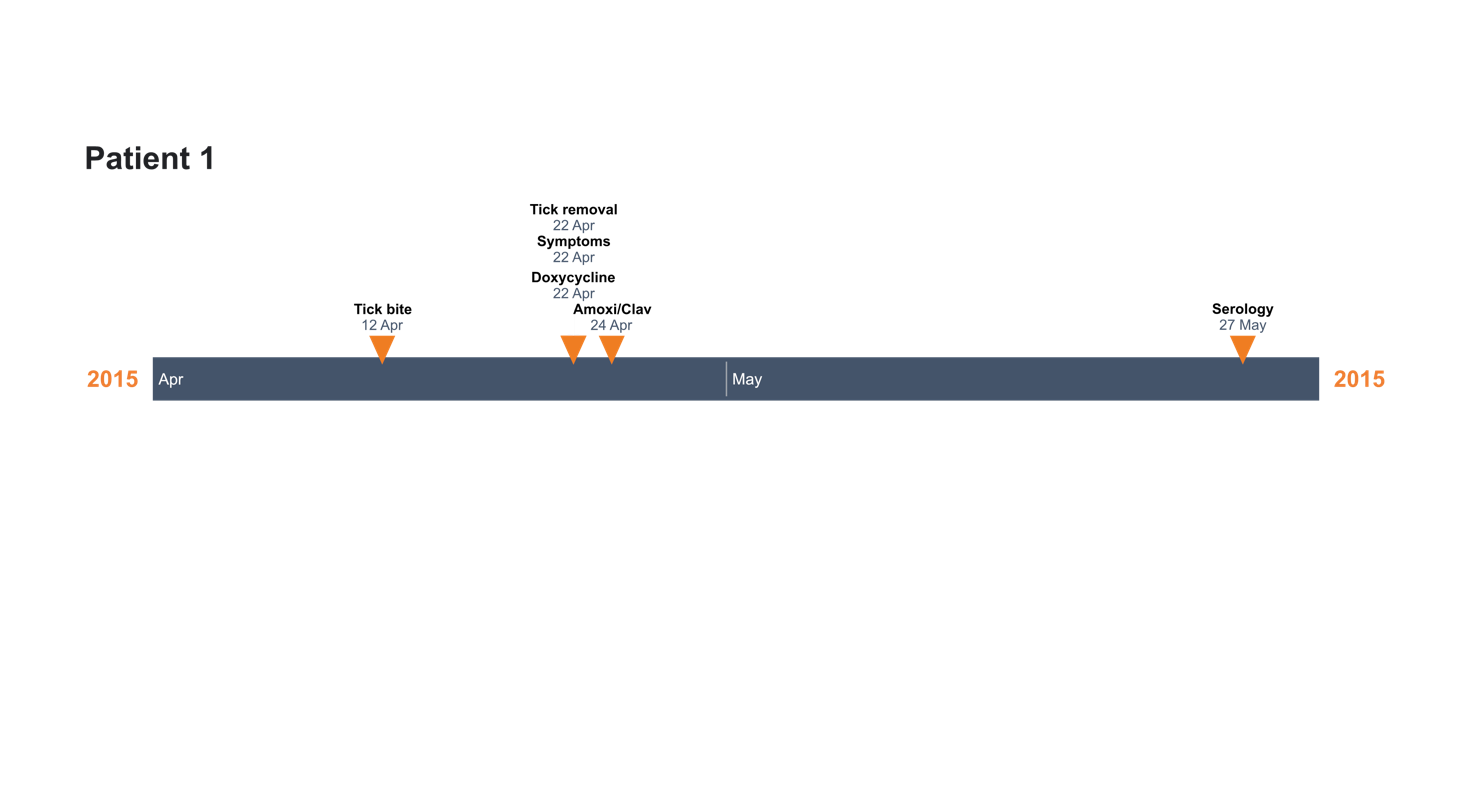


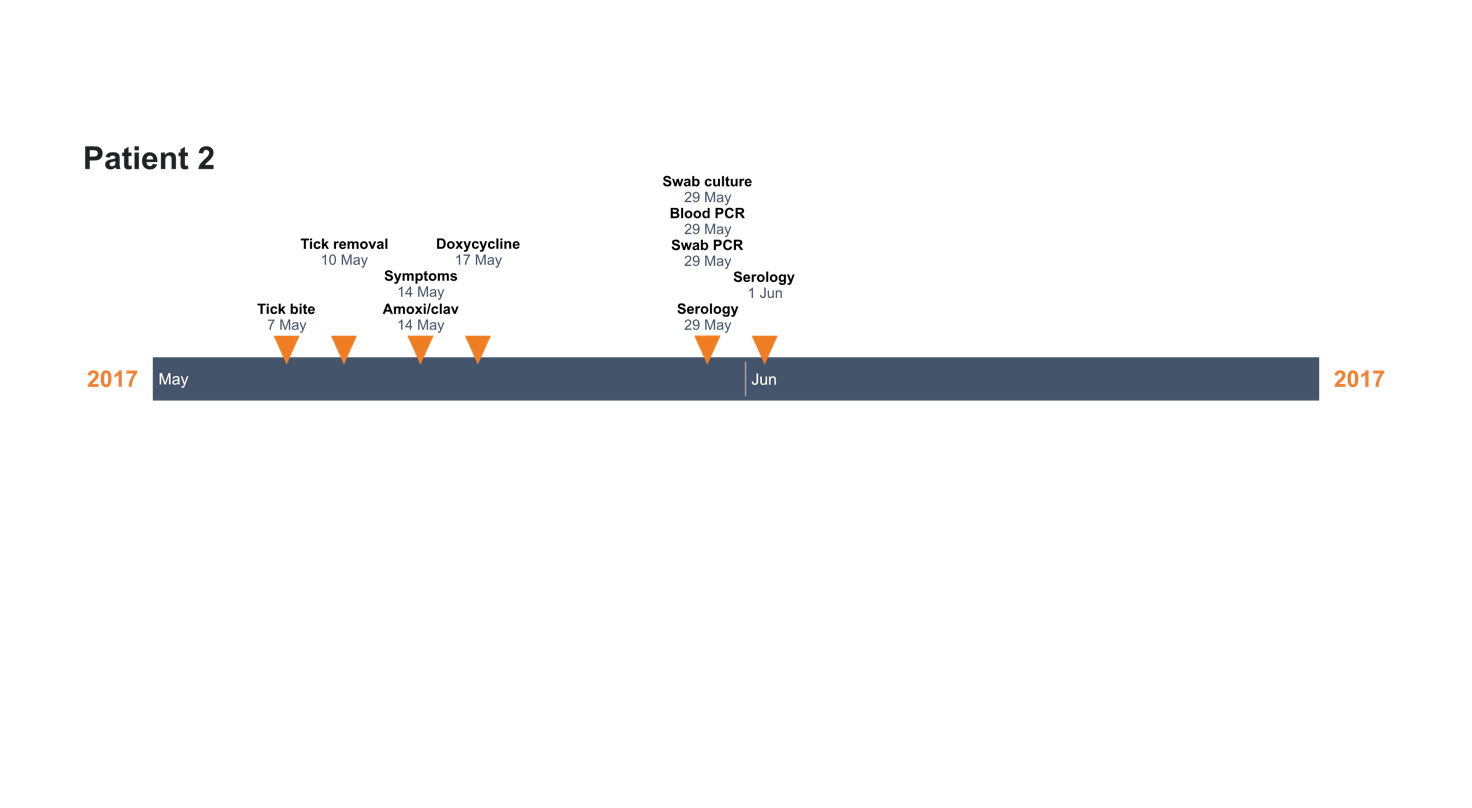


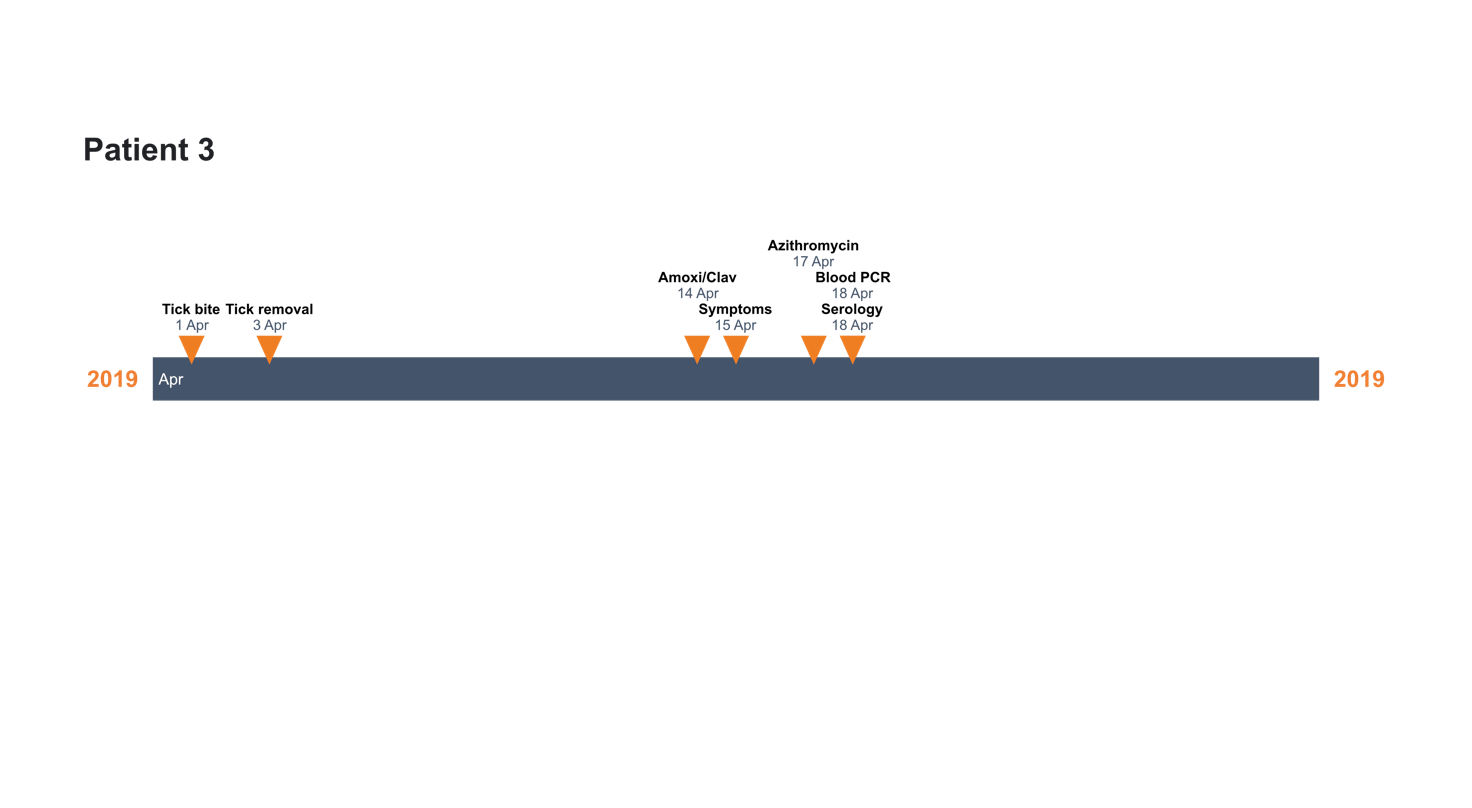


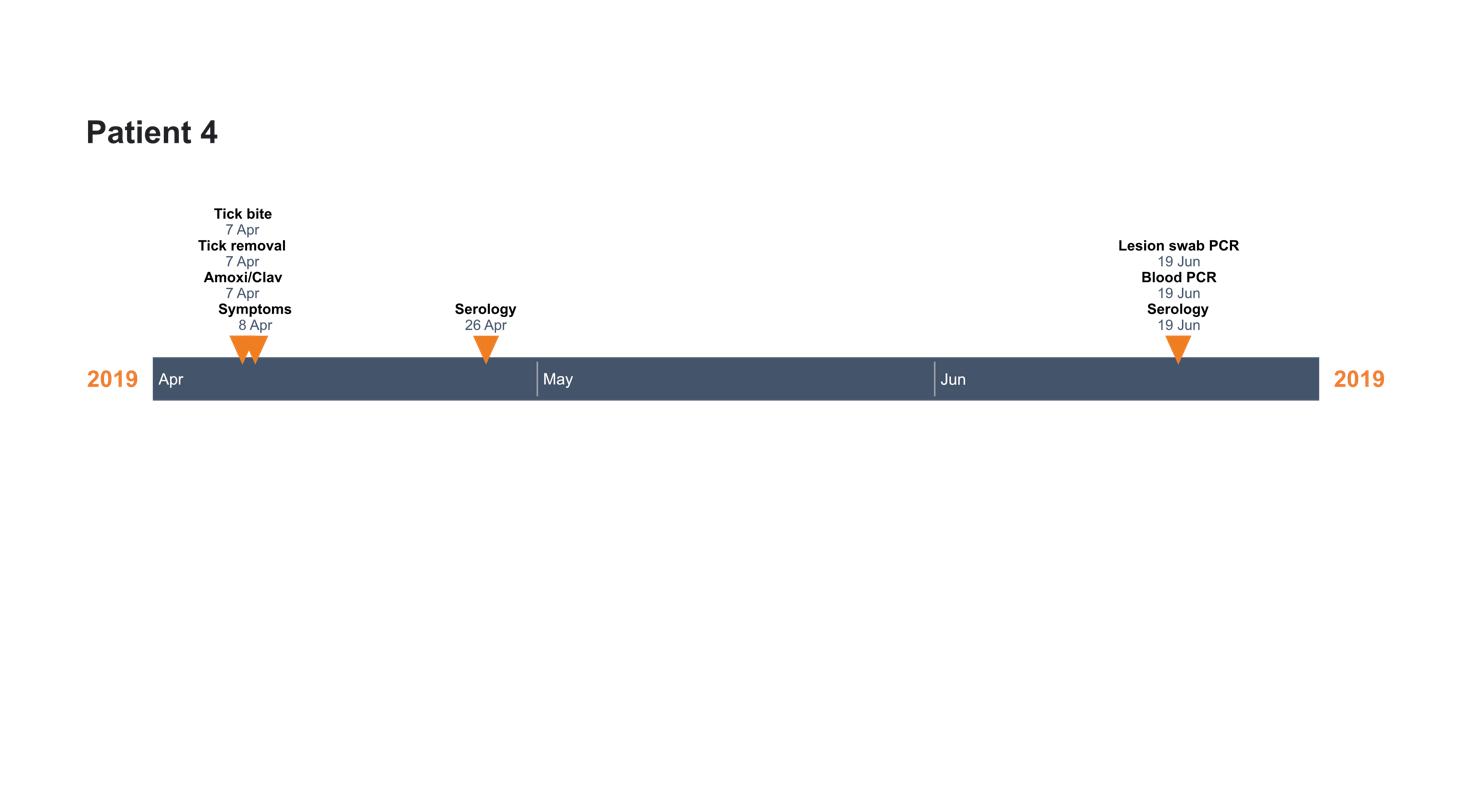


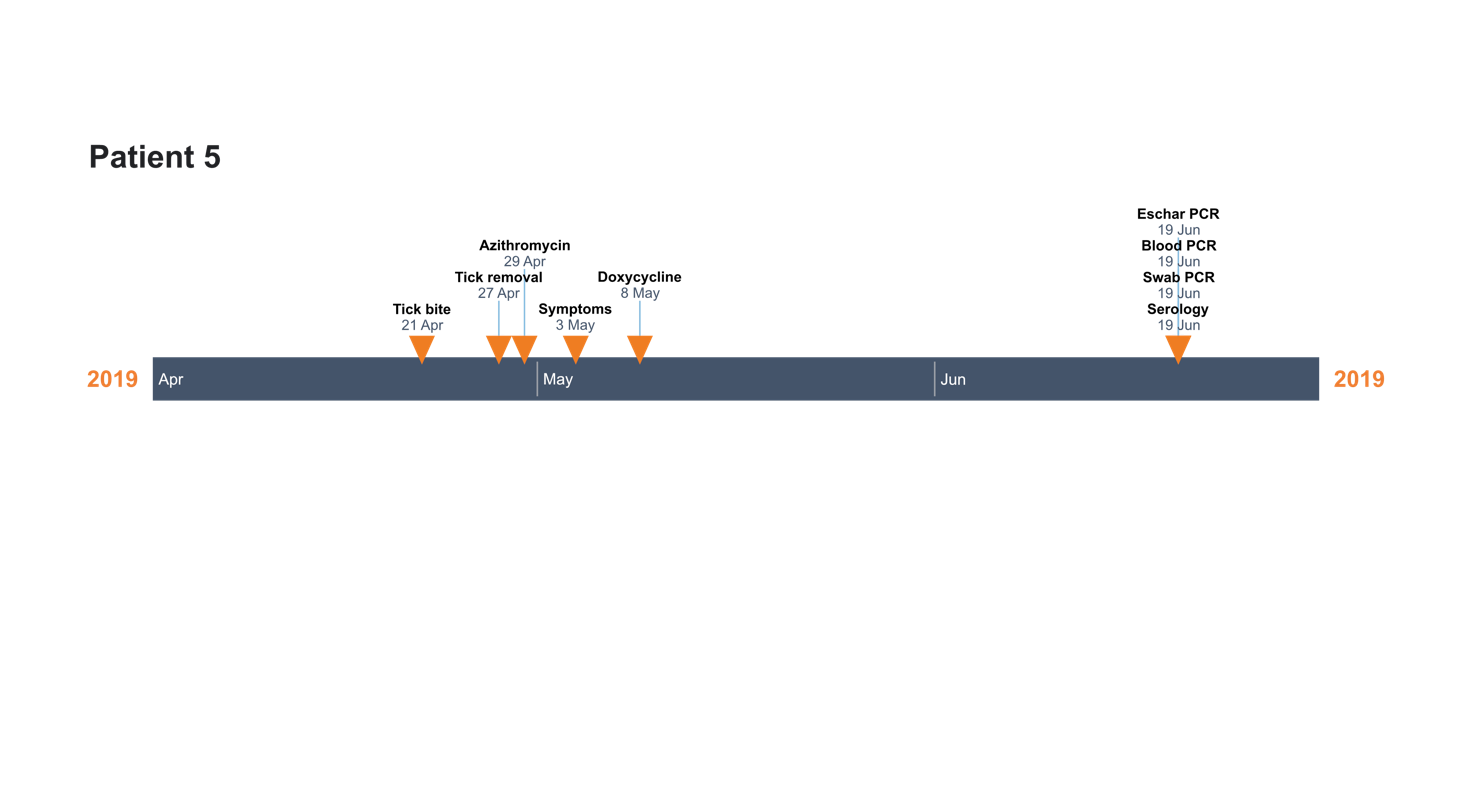


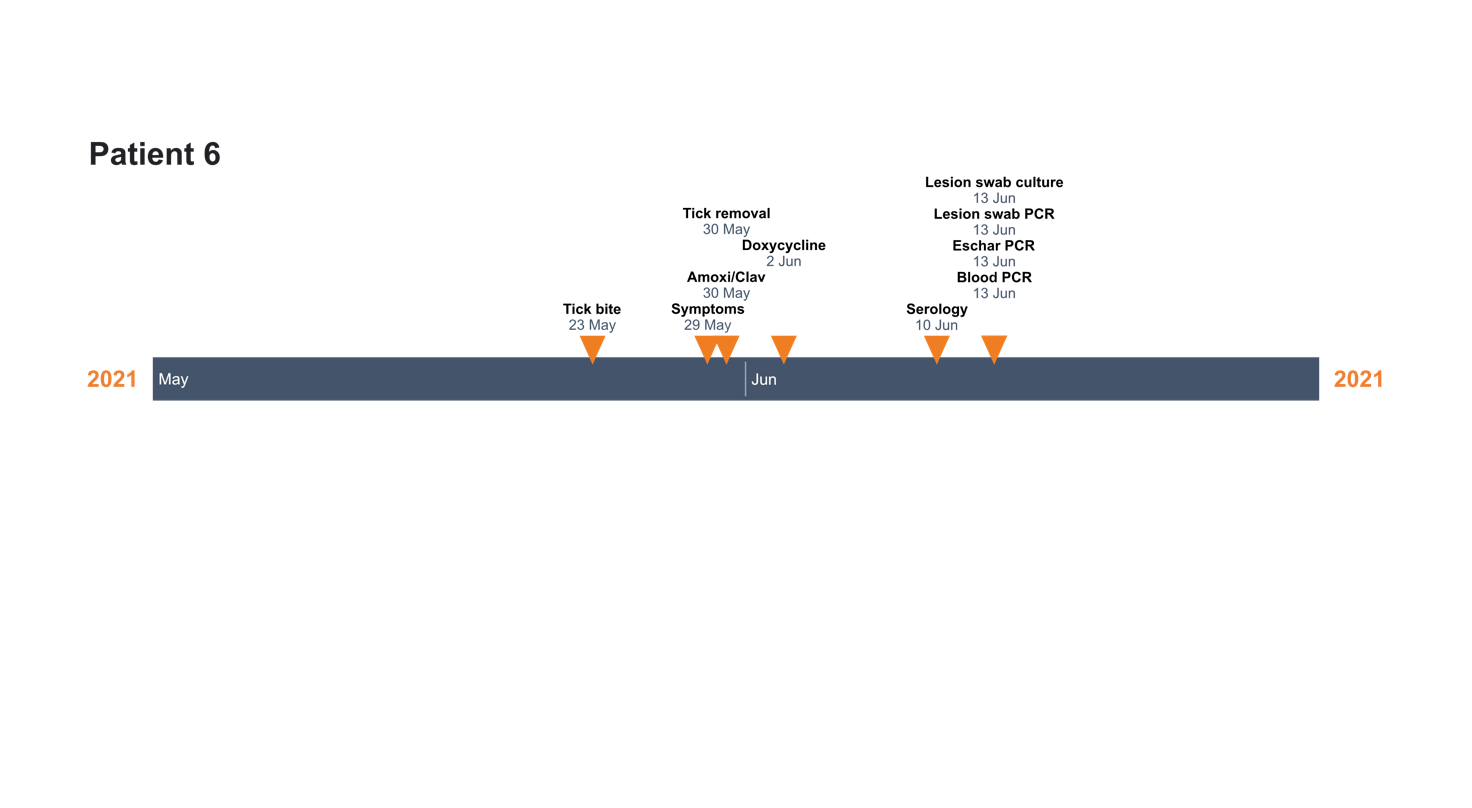

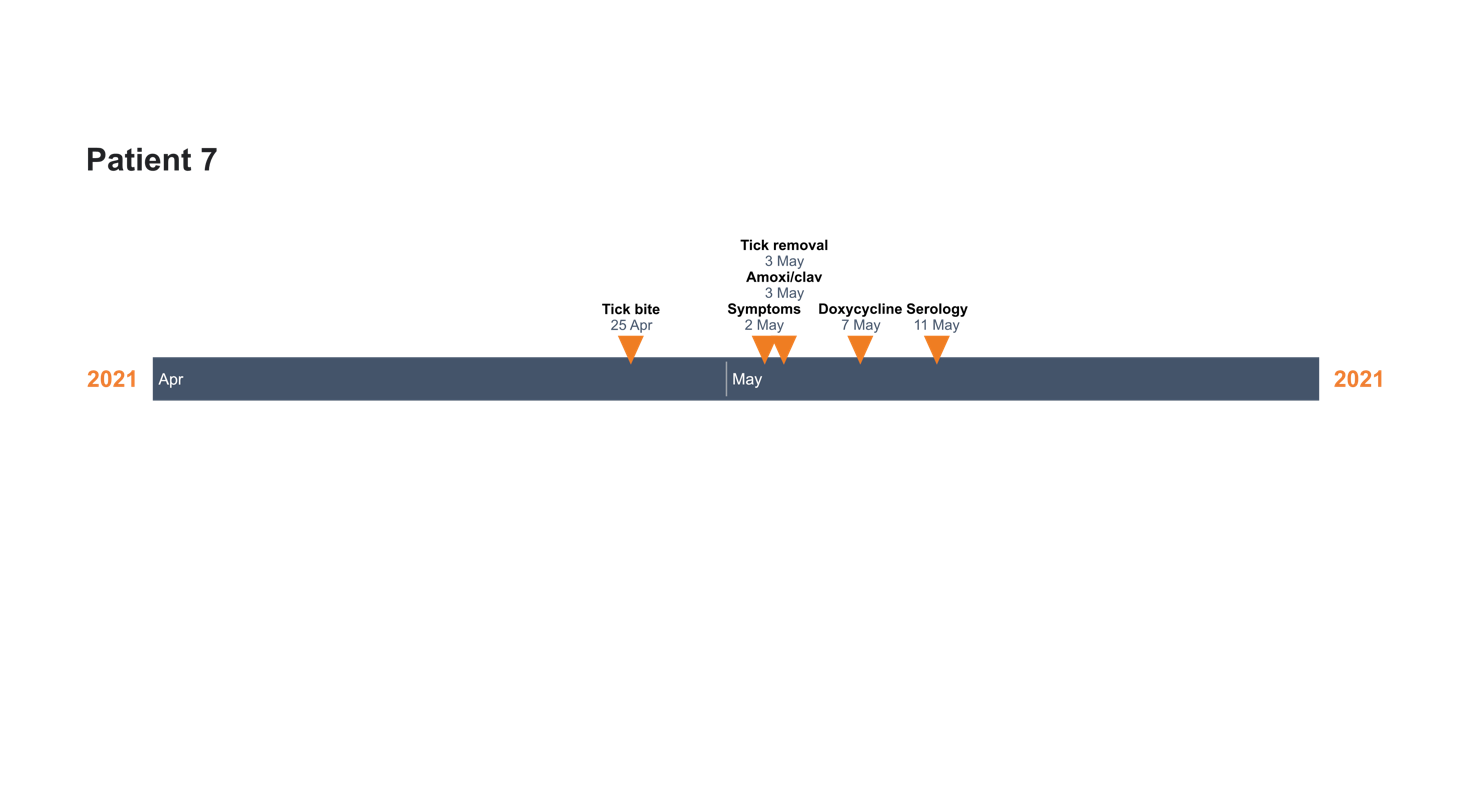

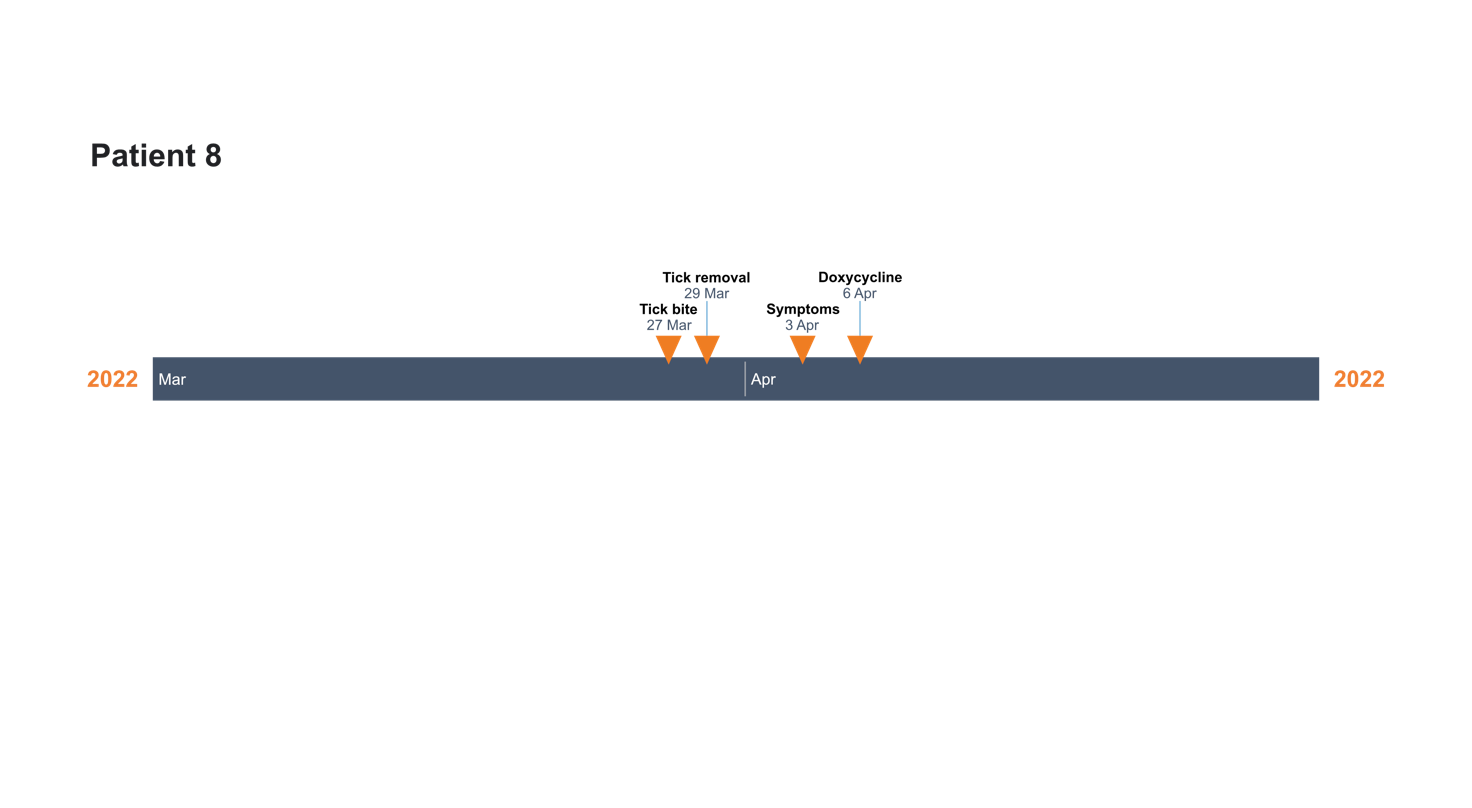


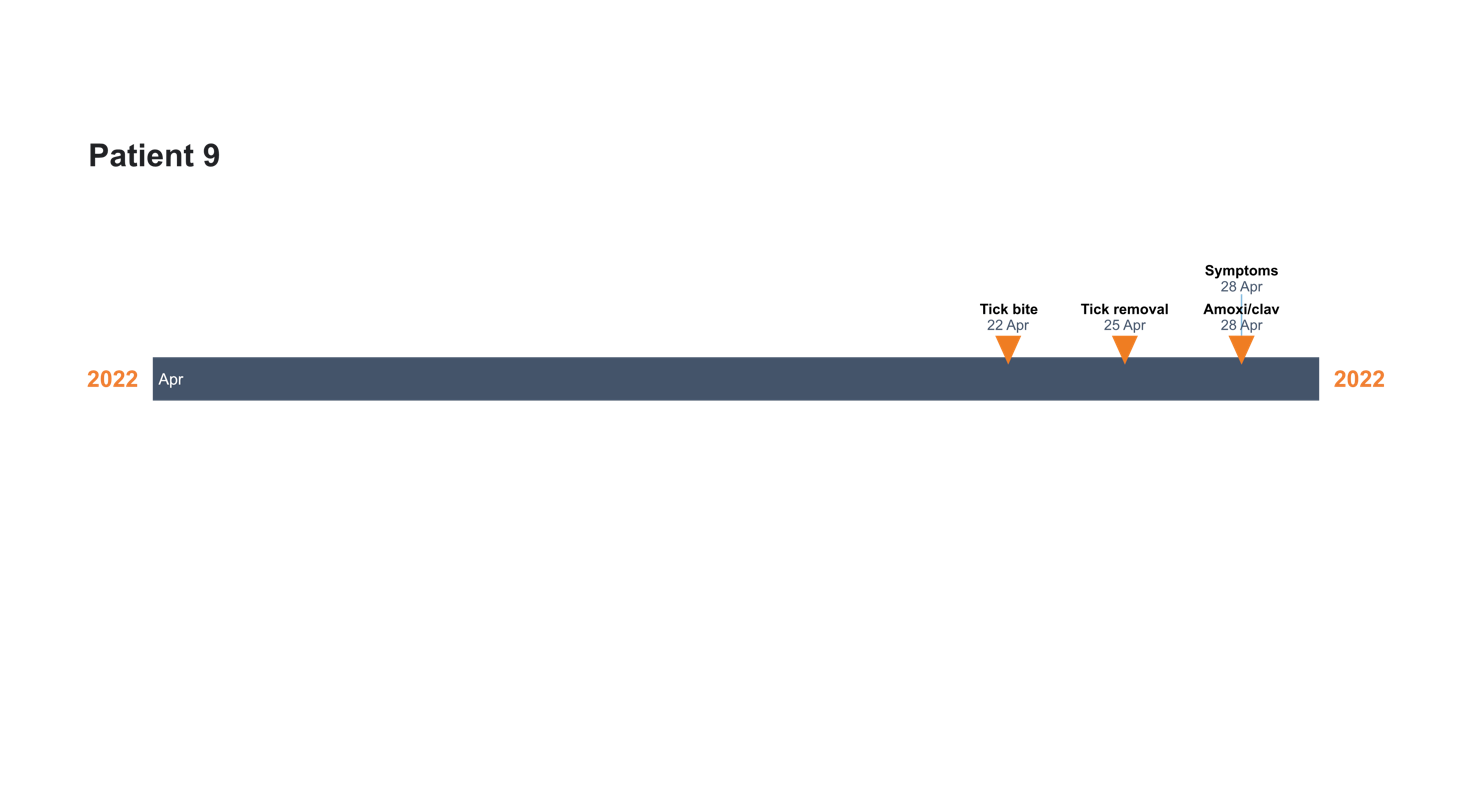


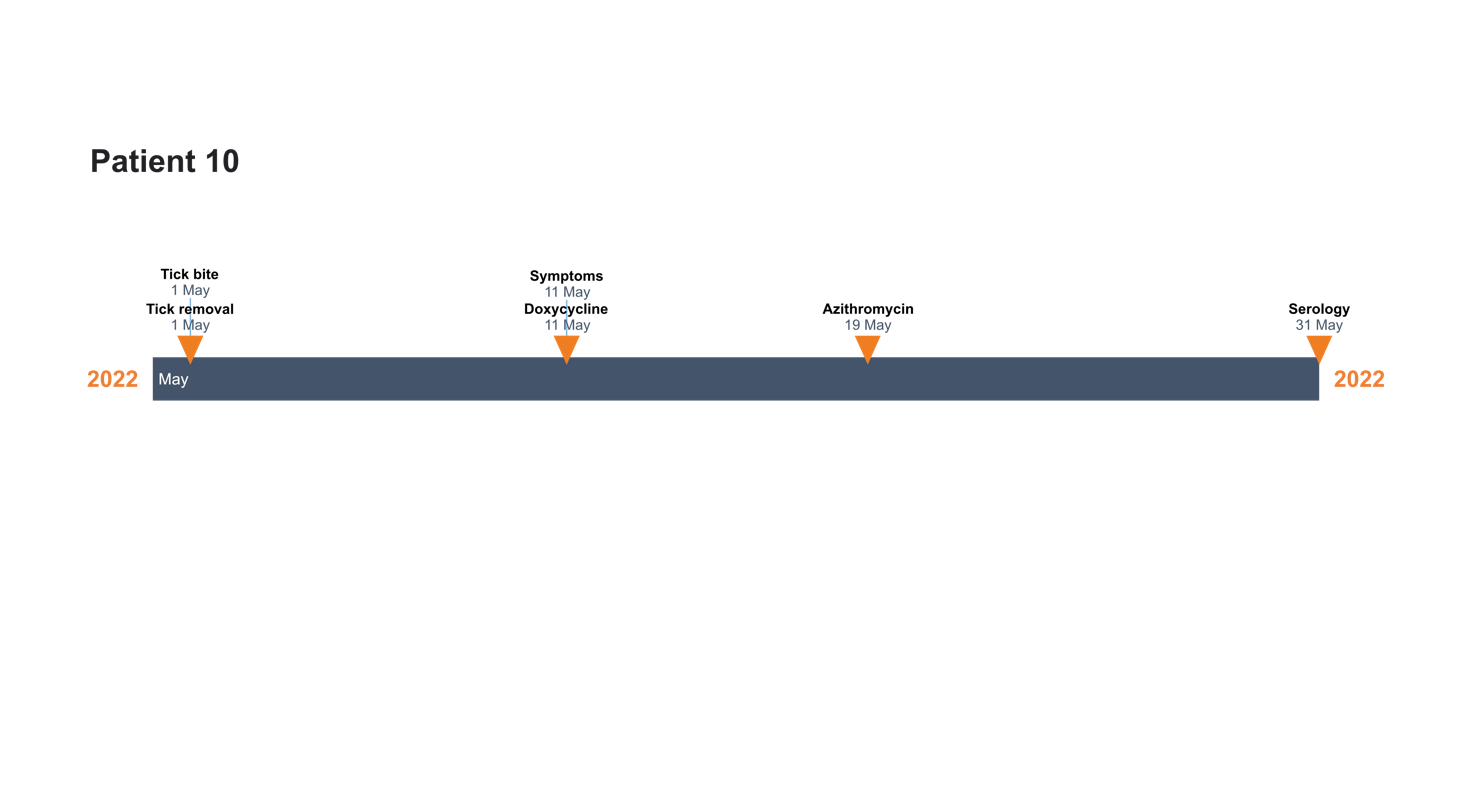


**Fig2:** Timelines representing main events (symptoms onset, treatment initiation, microbiological tests) are showed for all patients.
